# Gynecological and Obstetrical Society of Baltimore

**Published:** 1891-07

**Authors:** W. S. Gardner

**Affiliations:** Sec’y.; 712 N. Howard St.


### GYNECOLOGICAL AND OBSTETRICAL SOCIETY OF BALTIMORE.

---

### APRIL MEETING.

---

The President, Dr. Henry M. Wilson, in the chair.

Dr. Wm. P. Chunn related a case of ascites, which he treated by tapping and permanent drainage with apparently good results.

Dr. B. B. Browne, operated more than a year ago upon a woman with ascites who also had an abdominal tumor which proved to be papillomatous. There has been no return of either the dropsy or the papillomatous growth. He referred to the many cases of laparotomy and washing out the abdominal cavity.

Dr. Geo. W. Mittenberger could not see why any malignant tumor should not be able by irritation of the serous membrane to cause ascites. We often see ascites without any definable cause and when a growth did exist, it seemed a very good reason for the presence of the fluid. He referred to the case of a colored woman operated upon by Dr. Neale.

Dr. L. E. Neale said that in the case of the colored woman referred to, there was no assignable cause for the ascites except the presence of a sub-serous uterine myomata; at the operation he removed the uterine appendages. The growth remained, but there was no return of the ascites. There was also a complete procedentia, but after the operation, he was enabled to keep the uterus in place with a soft rubber ring. The tumor gradually diminished and ultimately disappeared. Is the exposure and irritation of the serous membrane during the operation a sufficient explanation of such an alteration in its function when the apparent cause of the ascitic extension remains?

He thought the question eminently important and practical in its bearings, and that it required further elucidation.

Dr. Wilmer Brinton remarked that in a case of cirrhosis of the liver in a male patient, tapping for the ascites had been followed by a permanent opening which persisted until the patient's death, one month afterward.

Dr. J. Whitridge Williams, in referring to Dr. Mosby's remarks, said that the ascites accompanying papillomatous growths, was considered to be due in great part to direct exudation from the vessels of the growth—he also referred to tubercular peritonitis.

Dr. B. B. Browne exhibited a small tumor about the size of a large hickory-nut and apparently a fibroid, which he had removed from a point a little to one side of the median line and between the clitoris and urethra. It pressed on the urethra, interfering with micturition. The growth was easily shelled out and the patient did perfectly well. It was the first growth of the sort he had seen in that locality.

Dr. Neale related a case of imperforated rectum in a white male child, naturally born, at full term, of healthy parents. The child was puny, weighing only 5 3-4 lbs. at birth, and 1 inch within the anus the rectum was imperforate. Dr. T. Hanny operated upon the child when it was two and a half days old, very feeble and partly cyanosed. No anesthetic was used—anus was cut through, the perineal structures laid open, the coccyx removed, the rectum opened through its posterior walls just above the imperforate part and its mucous membrane stitched to the skin just behind the original aperture. The stitches sloughed out and the large wound healed slowly by granulation. A copious discharge of flatus and meconiun occurred during the operation and the tympanitic abdomen disappeared.

Profound shock and collapse followed the operation, the child lying motionless, the feet and lower limbs cyanosed, the face and head less so—jaw dropped, mouth opened, eyes closed, lids blue, surface temperature but little, if at all. lowered. No cry. The features were frequently pinched or wrinkled from pain, becoming more or less blue at irregular intervals.

In this condition the child would make no effort or motion, but would swallow two teaspoonfuls at a time of milk and brandy when poured into its mouth, rarely refusing to swallow and never vomiting the food and stimulus which were given freely and frequently.

For nearly two days and a half did it remain in this state, partially rousing during the administration of food or other disturbance, and again relaxing. Even after this period when the first decided improvement occurred, the child would frequently relapse and remain in this condition for hours at a time. The first two weeks of its life was passed in this manner. The digestive and urinary apparatus functioned normally.

From the tenth to the fourteenth day these attacks gradually diminished and ultimately disappeared.

The child is now nearly two months old, but very feeble, and weighs only 5 1-4 lbs. It has been reared chiefly on condensed milk. The dense cicatrix just about the seat of the old imperforation has to be dilated daily with the finger ; another operation will be necessary. No diagnosis of abnormality in vascular system could be made.

Dr. Brinton mentioned a case of a child which lived nine or ten days with an open ductus anteriosus.

He thought that no ordinary trouble could account for the symptoms in the case. The cyanosis would not clear up entirely and then recur. He did not consider the condition one of collapse. There was no feebleness of pulse or coldness of surface. The child would lie in an apparently comatose condition with no evidence of sensation and then recover. The first attack followed immediately the operation and evidently from shock; but after two or three days it could not be attributed to this cause. There was no chill or febrile condition.

After the child had commenced taking food he used quinine by inunction and also small doses of dyalized iron, and as he believes with benefit from the latter.

He was inclined to account for the condition in this way: A very feeble child had food forced upon it for eight or ten hours, and when it had taken in all it could it apparently fell into a condition similar to that of vomiting animals, and when the supply of food was exhausted it would recover and take more nourishment. This condition entirely disappeared after the first two weeks. W. S. GARDNER, M. D., Sec'y.

*712 N. Howard St.*

---

DEATH AFTER A DOSE OF SALOL.—Salol is usually considered a tolerably innocuous drug, but there are not wanting clinical observations which tend to show that, under certain circumstances at least, its use may be followed by dire results. Thus a case was some time ago reported by Aufrecht and Behm in which death followed its use in acute endocarditis, and more recently Dr. Chlapowski has published in a Bohemian medical journal an account of a case in which a similar fatal result followed a fifteen grain dose ordered to a patient who was suffering from severe gastric symptoms, and who was being examined by Ewald's method.—*Lancet.*

